# Autophagy inhibition enhances PD-L1 expression in gastric cancer

**DOI:** 10.1186/s13046-019-1148-5

**Published:** 2019-03-29

**Authors:** Xiaojuan Wang, William K. K. Wu, Jing Gao, Zhongwu Li, Bin Dong, Xiaoting Lin, Yilin Li, Yanyan Li, Jifang Gong, Changsong Qi, Zhi Peng, Jun Yu, Lin Shen

**Affiliations:** 1 Department of Gastrointestinal Oncology, Key laboratory of Carcinogenesis and Translational Research (Ministry of Education/Beijing), Peking University Cancer Hospital and Institute, Beijing, 100142 China; 2 Department of Anaesthesia and Intensive Care, The Chinese University of Hong Kong, Hong Kong, China; 3 Department of Pathology, Peking University Cancer Hospital and Institute, Beijing, China; 4 Central Laboratory, Peking University Cancer Hospital and Institute, Beijing, China; 5 Department of Medicine and Therapeutics, The Chinese University of Hong Kong, Hong Kong, China

**Keywords:** Autophagy, PD-L1, Gastric cancer, NF-κB

## Abstract

**Background:**

Autophagy, a process for degrading intracellular substances to maintain basal metabolic turnover, is known to be perturbed in gastric cancer. Programmed cell death-1 (PD-1) with its ligand (PD-L1) are important immune checkpoint proteins and their regulation by autophagy has been reported in mouse melanoma and human ovarian cancer. Here, we explored the interplay between autophagy and the PD1/PD-L1 axis in gastric cancer.

**Methods:**

The expression of PD-L1 in gastric cancer cells was detected by Western blot and flow cytometry analysis. The effect of autophagy inhibition on PD-L1 expression was examined in vitro and in vivo. The molecular mechanisms of the regulation of PD-L1 by autophagy were evaluated in gastric cancer cell lines. The clinical relevance of autophagy-related markers p62/SQSTM1 and LC3 with PD-L1 was evaluated in 137 patients with gastric cancer.

**Results:**

We found that inhibition of autophagy by pharmacological inhibitors or small interfering RNAs increased the levels of PD-L1 in cultured gastric cancer cells and in xenografts. Interferon (IFN)-γ also promoted PD-L1 gene transcription, whose action was enhanced by autophagy inhibition. Mechanistically, autophagy inhibition led to the accumulation of p62/SQSTM1 and activation of nuclear factor (NF)-κB, in which NF-κB inhibition or p62/SQSTM1 knockdown attenuated PD-L1 induction by autophagy inhibition. Immunohistochemical staining of primary tumor tissues of 137 patients with gastric cancer showed that LC3 and p62/SQSTM1 protein levels were positively correlated with PD-L1 (LC3, *p* < 0.001; p62/SQSTM1, *p* < 0.05). The expression of PD-L1 was also positively correlated with tumor lymphocyte infiltration (*p* < 0.001).

**Conclusions:**

We discovered that autophagy regulates PD-L1 expression in gastric cancer through the p62/SQSTM1-NF-κB pathway. Pharmacological modulation of autophagy may thus influence the therapeutic efficacy of PD-L1 blockade in gastric cancer.

**Electronic supplementary material:**

The online version of this article (10.1186/s13046-019-1148-5) contains supplementary material, which is available to authorized users.

## Background

Gastric cancer is a prevalent malignant tumor of the digestive tract and remains the third leading cause of cancer-related death worldwide. An estimated 951,600 newly diagnosed cases and 723,100 deaths of gastric cancer occurred in 2012 [1]. The 5-year survival rate for patients with advanced gastric cancer is only 5–20% with 10 months of median overall survival [1]. Therefore, new molecular targets and therapeutic approaches are urgently needed.

Autophagy is a highly conserved homeostatic process that involves the formation of a double-membrane structure, autophagosome, which subsequently fuses with the lysosome to generate autolysosome leading to the degradation of the cellular proteins and damaged organelles. This catabolic pathway plays a pivotal role in cell survival, cellular metabolism and immune responses. Growing evidence reveals that the role of autophagy in tumorigenesis is complex and context-dependent [2]. On one hand, autophagy can inhibit tumor formation by reducing oxidative stress and DNA damage in normal tissues [3]. On the other hand, autophagy can promote tumor cell survival by providing cells with energy and vital compounds upon various stress stimuli in developed cancers [2, 4]. Also, autophagy can be activated in response to cytotoxic chemotherapeutics, acting as a mechanism of drug resistance [5–7]. Thus, modulating autophagy is an attractive option, which allows us to harness this process for improving the disease management in cancers.

Programmed cell death-1 (PD-1) with its ligand (PD-L1) are important immune checkpoint proteins. Elevated expression of PD-L1 receptors on cancer cell membranes has been observed in many cancer types. PD-L1 can interact with PD-1 and CD80 on the surface of T-cells, thus protecting cancer cells from immune-mediated rejection by inhibition of T effector functions [8, 9]. PD-L1 expression can be induced by inflammatory cytokines, such as interferon (IFN)-γ [10] secreted by infiltrating lymphocytes or induced by tumor-cell intrinsic signaling, including nuclear factor (NF)-κB, mitogen-activated protein kinase (MAPK), phosphoinositide 3-kinase (PI3K), mammalian target of rapamycin (mTOR) and Janus kinase/signal transducers and activators of transcription (JAK/STAT) [11]. In addition, PD-L1 is regulated by the tumor suppressor genes *PTEN* and *LKB1* as well as epithelial-mesenchymal transition-related molecules [12, 13]. More recently, evidences suggest that PD1 receptor and its ligand PD-L1 can have crosstalk with autophagy in cancer cells. In mouse melanoma and human ovarian cancer, tumor cell-intrinsic PD-L1 upregulates mTOR complex 1 signaling to inhibit autophagy and sensitizes tumor cells to clinically available autophagy inhibitors [14]. Recent work shows that CMTM6 co-localizes with PD-L1 at the cell membrane and in endosome, where it protects PD-L1 from lysosome-mediated degradation in a broad range of cancer cells [15]. Defective autophagy has also been shown to promote PD-L1 expression in cerulein-treated Atg5^L/L^ mice with pancreatitis [16]. The link between autophagy and PD-L1 in gastric cancer is unclear. Here, we investigated if tumor-intrinsic PD-L1 could be regulated by autophagy in gastric cancer. To test our hypothesis, we determined if inhibition of autophagy could increase PD-L1 levels in human gastric cancer cells.

## Methods

### Gastric cancer cell lines

Eight gastric cancer cell lines (AGS, BGC823, HGC27, MGC803, MKN28, MKN45, NCI-n87 and SGC7901) and a human normal gastric epithelial cell line (GES-1) were used in this study. Cell lines were maintained in RPMI-1640 medium or DMEM medium with 10% fetal bovine serum.

### Human sample collection

One hundred and thirty-seven primary gastric cancer samples were collected during surgical resection at Peking University Cancer Hospital in Beijing, China. None of these patients received preoperative chemotherapy or radiotherapy. The diagnoses of gastric cancer were all histologically confirmed and all subjects provided informed consent for obtaining the study specimens. The study protocol was approved by the Clinical Research Ethics Committee of Peking University Cancer Hospital and Institute.

### Reagents, antibodies and commercial kits

RPMI1640 medium (72400) and DMEM medium (10564) are products from Life Technologies. 3-methyladenine (M9281), bafilomycinA1 (B1793), chloroquine (C6628), rapamycin (R0395) and phytohemagglutinin-M (PHA, L8902) are from Sigma-Aldrich. BMS 345541 (S8044) is from Selleck. The following primary antibodies were used: microtubule-associated light chain 3 (LC3B, NB100–2220, Novus Biologicals), LC3A/B (13,082, Cell Signaling), p62/SQSTM1 (H00008878-M01, Novus Biologicals), PD-L1 (NBP1–76769, Novus Biologicals), PD-L1 (59,949, Cell Signaling), PD-L1 (Spring Bio, SP142), ATG5 (12,994, Cell Signaling), ATG7 (SAB4200304, Sigma-Aldrich), β-actin (4967, Cell Signaling), CD45 (368,508, Biolegend), CD8a (301,041, Biolegend), CD4 (357,408, Biolegend), FITC Mouse IgG1(400,110, Biolegend), PD-L1 (329,708, Biolegend), APC Mouse IgG2b (300,907, Biolegend), and 7-AAD (420,404, Biolegend).

### RNA interference

The expression of ATG5, ATG7, PD-L1, SQSTM1 and p65 was lowered using target-specific small interfering RNA (siRNA) molecules purchased from Qiagen as follows: Control siRNA (SI03650318), ATG5 siRNA (SI02655310), ATG7 siRNA (SI02655373), PD-L1 siRNA (SI03093076, SI03021158, SI00103250, SI00103243), SQSTM1 siRNA (SI00057596), RELA siRNA (SI02663101, SI02663094, SI00301672, SI05146204). Two hundred picomoles of gene-specific or control siRNA was transfected into cells at 40–60% confluence using Lipofectamine™ 3000 reagent (Invitrogen, 30,000–15) according to the manufacturer’s instructions.

### Animal experiments

MKN45 cells (1 × 10^7^ cells in 0.1 ml phosphate-buffered saline) were injected subcutaneously into the dorsal left flank of 4-week-old male BALB/c nude mice (*n* = 5 per group). Tumor diameter was measured every 2 days for 3 weeks. Pharmacological modulation of autophagy was achieved by intraperitoneal administration of chloroquine (50 mg/kg) every other day for 3 weeks. Tumor volume (mm^3^) was estimated by measuring the longest and shortest diameter of the tumor and calculating as previously described. All experimental procedures were approved by the Animal Ethics Committee of Peking University Cancer Hospital and Institute.

### Lymphocyte preparation

Peripheral blood mononuclear cells (PBMC) were isolated from heparinised peripheral blood samples obtained from gastric cancer patients by Ficoll-Paque (GE Healthcare Life Sciences) density gradient centrifugation. To induce production of PD-1, PBMC were resuspended in RPMI-1640 containing 5 mg/mL PHA, 5% heat inactivated human AB serum, 1% penicillin, streptomycin, and amphotericin (Gibco), and incubated for 48 h [17]. This PBMC culturing method was used to induce proliferation of activated T lymphocytes by mitogen activation and precondition them to express PD-1. Then cells were rested overnight in the same growth condition minus the PHA. These cells were then co-cultured with the gastric cancer cells.

### Drug treatment of melanoma cells and coculture with lymphocytes

Gastric cancer cells were plated on two sets in 12-well plates, and on the following day, treated either with DMSO, CQ (chloroquine), 3-MA (3-methyladenine), Baf (bafilomycin A1) or Rap (rapamycin). After another 24-h period, one set was treated with media and the other set with INF-γ. The final concentrations of the drugs were 16 μmol/L for CQ, 10 mmol/L for 3-MA, 10 nmol/L for Baf, 100 nmol/L for Rap, and 200 U/mL for INF-γ. In the case of cocultures, all the steps and conditions were the same, and on the next day, a suspension of lymphocytes (primed as described in the above) was added to each well. The final concentration of lymphocytes was 550,000 cells/mL. Each assay was repeated at least twice.

### Histology and immunohistochemical staining

Formalin-fixed and paraffin-embedded blocks were sectioned at 5 μm and stained with hematoxylin and eosin. Immunohistochemistry was performed on paraffin sections of gastric cancer tissues using anti-LC3B antibody (1:2000), anti-p62/SQSTM1 antibody (1:2000) or anti-PD-L1 antibody (1:100). The immunostaining score was estimated based on the positive cell and the staining intensity, as described previously [18]. The percentage of positively stained cells was graded as follows: grade 0, < 5%; grade 1, 5–25%; grade 2, 25–50%; grade 3, > 50%. Immunostaining intensity was rated as follows: 0, negative; 1, weak; 2, moderate; and 3, strong. The total expression score was the product of the aforementioned factors, which ranged from 0 to 9. The expression was grouped into low expression (scores of 0–3) and high expression (scores of 4–9).

### Real-time PCR

RNA was extracted using Trizol reagent (15596–026, Life technologies) and reverse-transcribed using SuperScript® III Reverse Transcriptase (18080–093, Life technologies). Real-time PCR (Applied Biosystems 7500 Fast Real-Time PCR System, Life technologies) was performed using the Power SYBR® Green PCR Master Mix with gene-specific primers: PD-L1, 5′-CAATGTGACCAGCACACTGAGAA-3′ and 5′-GGCATAATAAGATGGCTCCCAGAA-3′.

### Flow cytometry

Gastric cancer cells were probed with phycoerythrin-conjugated anti-PD-L1antibody. In the coculture experiments to distinguish gastric cancer cells from the immune cells, samples were stained with both anti-PD-L1 and anti-CD45 (Fig. 5a). All live/dead discrimination was performed with 7-aminoactinomycin D (7AAD). Lymphocytes were stained with CD45, CD8a, and CD4. All samples were run on a BD Accuri™ C6 Plus flow cytometer. Cells were gated according to the following schema: morphology was determined by using the area of the forward scatter emission peak (FSC-A) versus the area of the side scatter emission peak (SSC-A). Segregation of single cells was determined using SSC-A versus the width of the side scatter emission (SSC-W). Comparing the 7AAD with the APC emission peak allowed analysis of PD-L1 for the gastric cancer cells. For coculture assays, live/dead and lymphocyte discrimination was determined by comparing area of the 7AAD emission peak with the area of the CD45 emission peak and then 7AAD with APC for gastric cancer cell. Median fluorescent intensity (MFI) of PD-L1 was taken from plots of PD-L1.

### Statistical analysis

The results are expressed as mean ± standard deviation (SD). Differences between two groups were compared by the Mann-Whitney U test or Student’s t test where appropriate. Multiple group comparisons were made by the Kruskal-Wallis test or one-way analysis of variance (ANOVA) where appropriate. The χ^2^ test was used for comparison of patient characteristics and distributions of expression and covariates by vital status. Crude relative risks (RRs) of death associated with expression of autophagy markers and other predictor variables were estimated by univariate Cox proportional hazards regression model. The difference in tumor growth rate between the two groups of nude mice was determined by repeated-measures analysis of variance. *P* values < 0.05 were taken as statistically significant.

## Results

### PD-L1 expression in gastric cancer cells

**Fig. 1:**
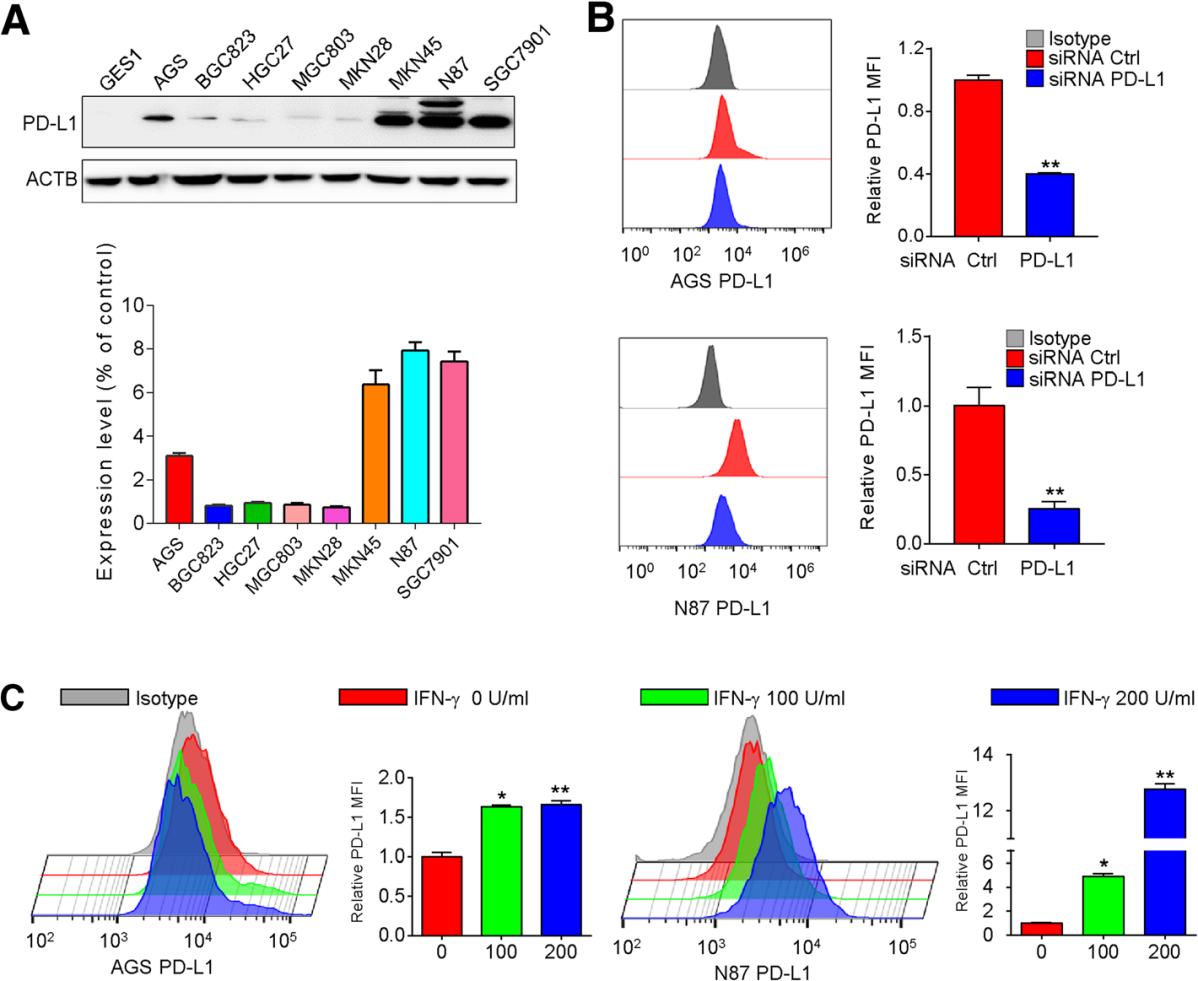
PD-L1 expression was evaluated in gastric cancer cells. **a** Levels of PD-L1 protein were assessed in normal human gastric epithelial cells and 8 gastric cancer cell lines by Western blots. **b**-**c** Validation of the anti-PD-L1 antibody by detecting the reduction of this ligand after siRNA knockdown (**b**) and induction of this ligand in the presence of INF-γ (**c**). Histograms showed PD-L1 levels on the surface of AGS and NCI-n87 cells in the mentioned conditions by flow cytometry. The ratio of PD-L1 MFI minus isotype control was shown as mean ± S.D. relative to Ctrl from 3 independent experiments, *p < 0.05, **p < 0.01

The protein levels of PD-L1 in 8 gastric cancer cell lines and a human normal gastric epithelial cell line (GES1) were first determined by Western blots and flow cytometry (Fig. 1a and Additional file 1: Figure S1). Protein expression of PD-L1 was variable among gastric cancer cell lines, showing a range of more than 10-fold difference (Fig. 1a). Of note, the majority of cell lines showed very low levels of PD-L1 expression. The validity of the anti-PD-L1 antibody was evaluated by its ability to detect the decrease of PD-L1 levels after siRNA knockdown of this ligand and the increase in expression of PD-L1 upon treatment of two gastric cancer cell lines (AGS and NCI-n87) with IFN-γ. Using the optimal titration of the antibody, flow cytometry assay indicated around 25 to 50% reduction of MFI by the PD-L1 siRNA pool in comparison with the non-targeted control siRNA pool in AGS and NCI-n87 cells (Fig. 1b). Induction of MFI by IFN-γ by about 50% was observed in AGS and 3 fold in NCI-n87 cells (Fig. 1c).

### Pharmacological blockade of autophagy upregulated PD-L1 expression in gastric cancer

**Fig. 2:**
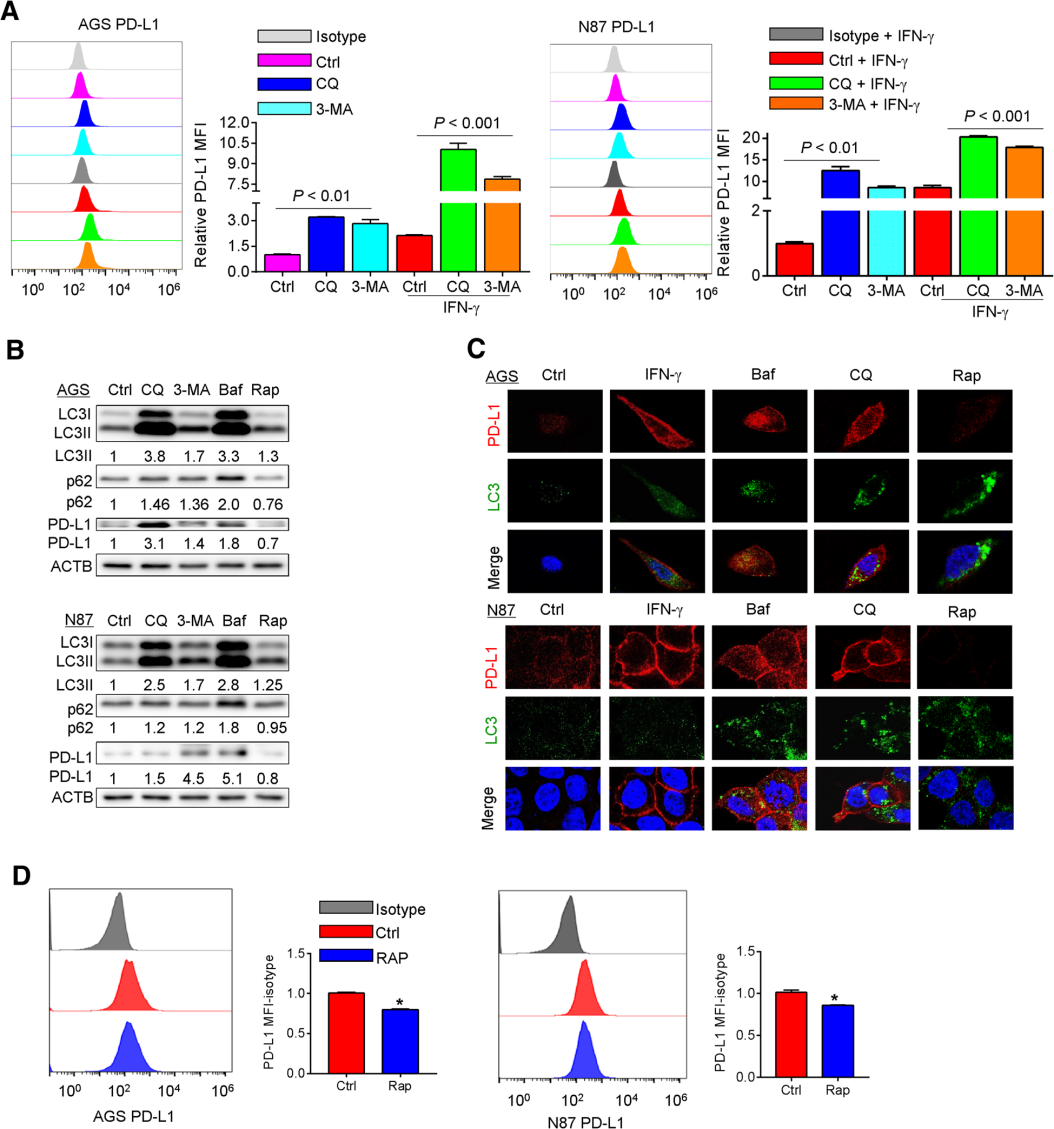
Effects of autophagy inhibitors in combination with IFN-γ on expression of PD-L1 in gastric cancer cell lines. **a** The effect of chloroquine (CQ) or 3-MA on expression of PD-L1 with or without INF-γ for 24 h was determined by flow cytometry assays. In AGS and NCI-n87 cells, MFI as the indication of PD-L1 expression level can be further increased by the treatment of INF-γ. **b** Levels of LC3B-I/II, p62/SQSTM1 and PD-L1 were determined by Western blots in AGS and NCI-n87 cells treated by CQ, 3-MA, bafilomycin A1 (Baf) or rapamycin (Rap) for 24 h. **c** Positive staining of PD-L1 (red) and LC3 positive puncta (green) was determined by immunofluorescence in AGS and NCI-n87 cells treated by autophagy inhibitors and activator as in (**b**). **d** Rapamycin decreased the levels of PD-L1 protein in AGS and NCI-n87 cells as shown by flow cytometry. Results were averaged and blots were representative of 4 independent experiments, *p < 0.05, **p < 0.01

The expression of PD-L1 is mainly regulated by IFN-γ through the activation of the JAK/STAT pathway, or by NF-κB, MAPK, PI3K and mTOR signaling [19, 20]. To investigate the potential effect of autophagy on PD-L1 expression, pharmacological inhibitors, including 3-MA at 10 mM, chloroquine at 32 μM and bafilomycin A1 at 10 nM, that blocks an early stage of autophagy or interferes with lysosomal function, were used to inhibit autophagy in two gastric cancer cell lines AGS and NCI-n87. To assess the efficacy of these inhibitors, the levels of LC3B-I and -II were determined. After lipidation from LC3B-I, LC3B-II translocates onto the surface of autophagosomal vacuoles, which is ultimately degraded by lysosomal enzymes in the autolysosomes. We found a markedly enhanced LC3B-positive puncta (Additional file 1: Figure S2) in AGS and NCI-n87 cells treated by chloroquine and bafilomycin A1, indicating lysosomal degradation of autophagosomes was impaired. The same treatments caused an induction of PD-L1 surface expression in AGS and NCI-n87 cells (Fig. 2a). Concordantly, in both cell lines, combination of chloroquine or 3-MA with IFN-γ caused a further increase in PD-L1 protein levels as compared with the groups treated by chloroquine or 3-MA alone (Fig. 2a). Upon treatment by the above-mentioned inhibitors, a significant induction in the protein expression of PD-L1 by Western blots was also observed (Fig. 2b). Increases in total LC3B-II and p62/SQSTM1 protein were observed in these cells upon autophagy inhibition. Increases of LC3B-II and decreases of p62/SQSTM1 protein were observed in these cells upon treatment by rapamycin (a known inducer of autophagy) at a dose of 100 nM (Fig. 2b). It is noteworthy that AGS and NCI-n87 cells showed a similar pattern of PD-L1 induction on the cell membrane upon addition of chloroquine and bafilomycin A1 whereas the levels of PD-L1 were decreases upon autophagy activation as shown by Western blots and immunofluorescence assay (Fig. 2b and c).

### Induction of autophagy by rapamycin reduced the levels of PD-L1 protein in gastric cancer cells

The data presented so far indicated an unreported connection between PD-L1 and autophagy, in which blockade of autophagy upregulated PD-L1. To assess if induction of autophagy could in reverse reduce the levels of PD-L1, AGS and NCI-n87 cells were treated with rapamycin. Upon treatment, autophagy was activated as shown by the increases in LC3B-II and decreases in p62/SQSTM1 proteins (Fig. 2b). Notably, PD-L1 was downregulated in AGS and NCI-n87 cells (Fig. 2b, c). The MFI of PD-L1 expression was decreased in rapamycin-treated AGS and NCI-n87 cells as compared to the control group (Fig. 2d).

### Knockdown of ATG5 and ATG7 prevented autophagy and up-regulated PD-L1 protein in gastric cancer cells

**Fig. 3:**
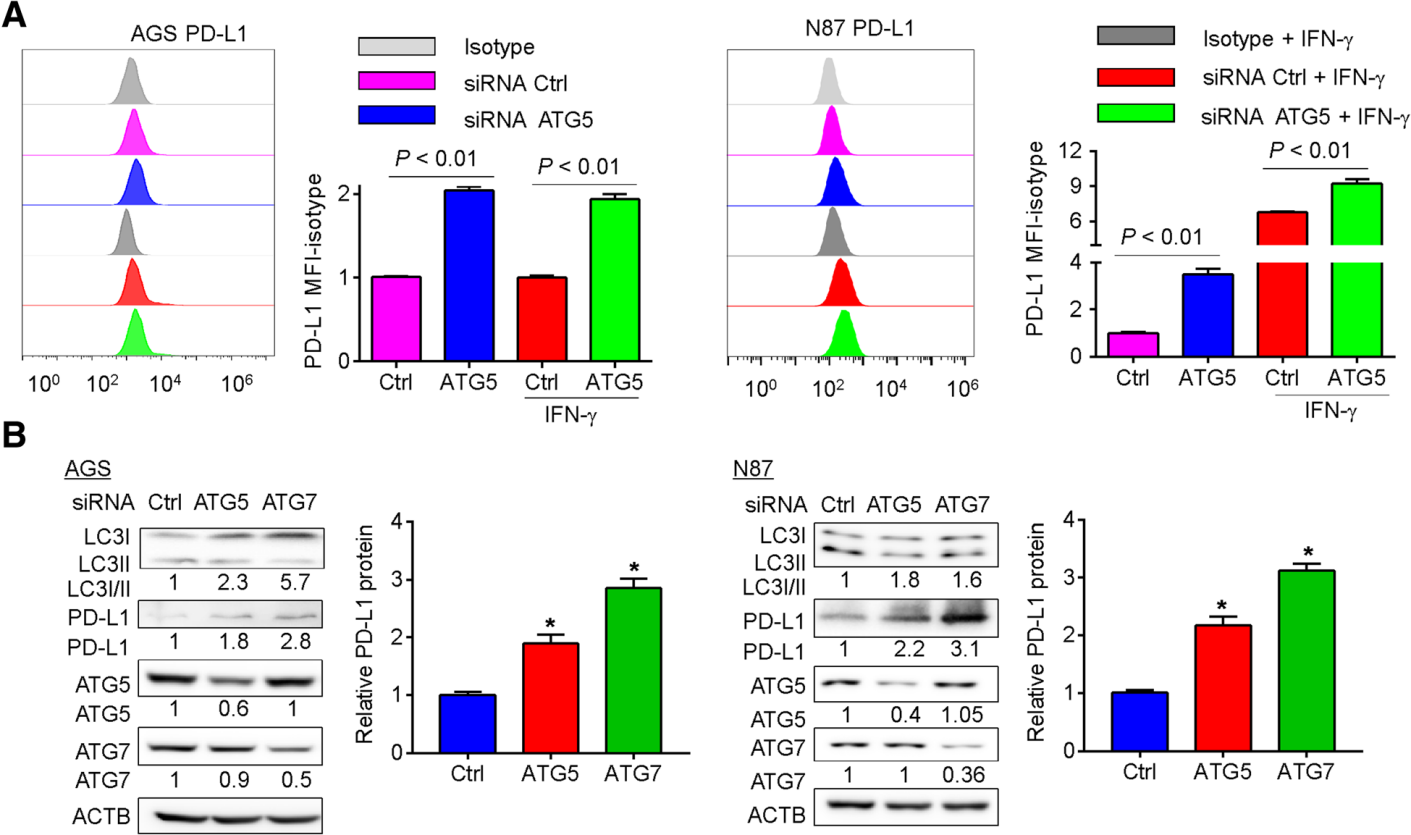
Abrogation of autophagy by siRNAs targeting autophagy-related genes in gastric cancer cells induced tumor-intrinsic PD-L1 expression. **a** Inhibition of autophagy by knockdown of ATG5 in AGS and NCI-n87 gastric cancer cells induced the expression of PD-L1 in the presence and absence of INF-γ (200 U/mL) as shown by flow cytometry analysis at 48 h post-transfection. **b** The induction of PD-L1 was confirmed by Western blots at 72 h post-transfection. The knockdown efficacies of ATG5 and ATG7 siRNA were verified. The conversion of LC3B-I to LC3B-II was reduced. Results were averaged and blots were representative of 4 independent experiments. The ratio of PD-L1 MFI minus isotype control was shown as mean ± S.D. relative to Ctrl from 4 independent experiments, *p < 0.05

In addition to pharmacological agents used in this study, siRNA targeting non-lysosomal components of autophagy was used to knock down proteins, namely ATG5 and ATG7, related to the autophagic machinery [21]. To assess if the inhibition of autophagy at the early stage could regulate PD-L1 expression, the levels of PD-L1 were determined in ATG5- and ATG7-depleted AGS and NCI-n87 cells. Similar to the effect of pharmacological inhibitors of autophagy, siRNA-mediated inhibition of autophagy increased the MFI of PD-L1 in both cell lines (Fig. 3a). A modest increase in PD-L1 protein level was also observed in ATG5-siRNA-transfected AGS and NCI-n87 cells in the presence of IFN-γ (Fig. 3a). The knockdown efficacies of ATG5- and ATG7-siRNAs were confirmed by Western blots. The efficacy of these siRNAs on autophagy inhibition was also determined by Western blots for LC3B-II. Unlike the blockade of autophagy at the late stage by chloroquine or bafilomycin A1, knockdown of ATG5 or ATG7 inhibited the conversion of LC3B-I to LC3B-II in AGS and NCI-n87 cells (Fig. 3b). As shown in Fig. 3b, knockdown of ATG5 or ATG7 upregulated the levels of PD-L1 protein. These findings suggested that inhibition of autophagy by knockdown of autophagy-related genes could increase tumor cell-intrinsic PD-L1 expression.

### Chloroquine prevented autophagy and induced PD-L1 in MKN45 mouse xenograft model

Because blockade of autophagy upregulated PD-L1 in AGS and NCI-n87 cells, we tested the effect of the pharmacological autophagy inhibitor chloroquine in a MKN45 mouse xenograft model. The tumor growth curves of chloroquine-treated or control mice are shown in Additional file 1: Figure S3A1. The tumor volume was slightly smaller in mice treated by chloroquine than in mice treated by solvent (*p* < 0.05, Additional file 1: Figure S3A2). Increased p62/SQSTM1 and LC3B-II protein levels were observed in mice treated with chloroquine (Additional file 1: Figure S3B), indicating that chloroquine inhibited autophagy in these mice. Consistent with the in vitro data, chloroquine treatment showed an inductive effect on the expression of tumor-intrinsic PD-L1 protein compared with control mice (Additional file 1: Figure S3B).

### Accumulation of p62/SQSTM1 and NF-κB activation upon autophagy inhibition contributed to the upregulation of PD-L1

**Fig. 4:**
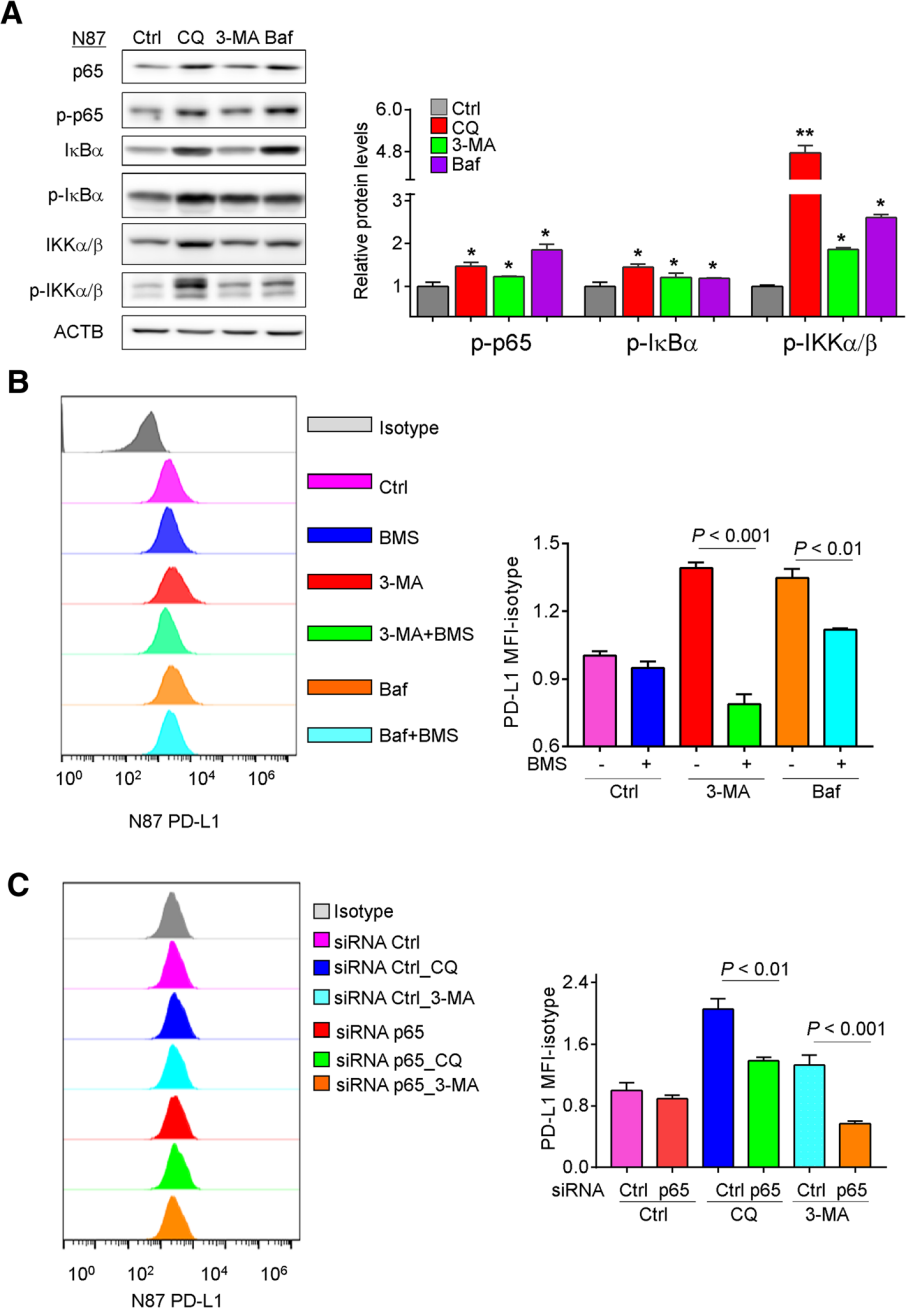
Autophagy inhibitors upregulated PD-L1 expression through NF-κB activation. **a** The effect of pharmacological inhibitors of autophagy on the protein levels of p65, p-p65, IκBα, p-IκBα, IKKα/β and p-IKKα/β was determined by Western blots in NCI-n87 cells. **b** NCI-n87 cells were treated with or without 3-MA and Baf for 24 h in the absence or presence of BMS-345541, and the levels of PD-L1 protein were examined by flow cytometry. **c** Knockdown of p65 alleviated the induction of PD-L1 expression at 72 h post-transfection in NCI-n87 cells upon autophagy inhibition by flow cytometry. Results were averaged and blots were representative of 3 independent experiment, *p < 0.05, **p < 0.01

PD-L1 expression in tumor cells could be triggered by intrinsic cellular signaling molecules, including NF-κB, MAPK, PI3K, mTOR and JAK/STAT [11]. The adaptor protein p62/SQSTM1 has been implicated in the activation of NF-κB pathway in many cell systems [22]. In this respect, when NCI-n87 and AGS cells were treated with pharmacological inhibitors of autophagy, upregulation of p65, phospho-p65, IκBα, p-IκBα, IKKα/β and p-IKKα/β proteins was observed, indicating NF-κB activation upon autophagy inhibition (Fig. 4a and Additional file 1: Figure S4A). In addition, inhibition of NF-κB signaling by its inhibitor, BMS-345541 (BMS, 2 μM), blocked the inducing effect of autophagy inhibitors on PD-L1 expression in NCI-n87 cells (Fig. 4b). However, the reversed effect of BMS-345541, an inhibitor of IκB Kinase, on the upregulation of PD-L1 by autophagy inhibition was not detected in AGS cells (Additional file 1: Figure S4B). Knockdown of p62/SQSTM1 expression by siRNA partially decreased the PD-L1 expression in AGS cells treated by chloroquine or bafilomycin A1 (Additional file 1: Figure S4C). Moreover, inhibition of NF-κB signaling by knocking down the expression of p65 decreased the levels of PD-L1 proteins in NCI-N87 and AGS cells treated by chloroquine or 3-MA (Fig. 4c and Additional file 1: Figure S4D). These results indicated that autophagy inhibitors upregulated the expression of PD-L1 by activation of NF-κB signaling.

### Effect of autophagy inhibitors on PD-L1 expression in cocultures of gastric cancer cells and lymphocytes

**Fig. 5:**
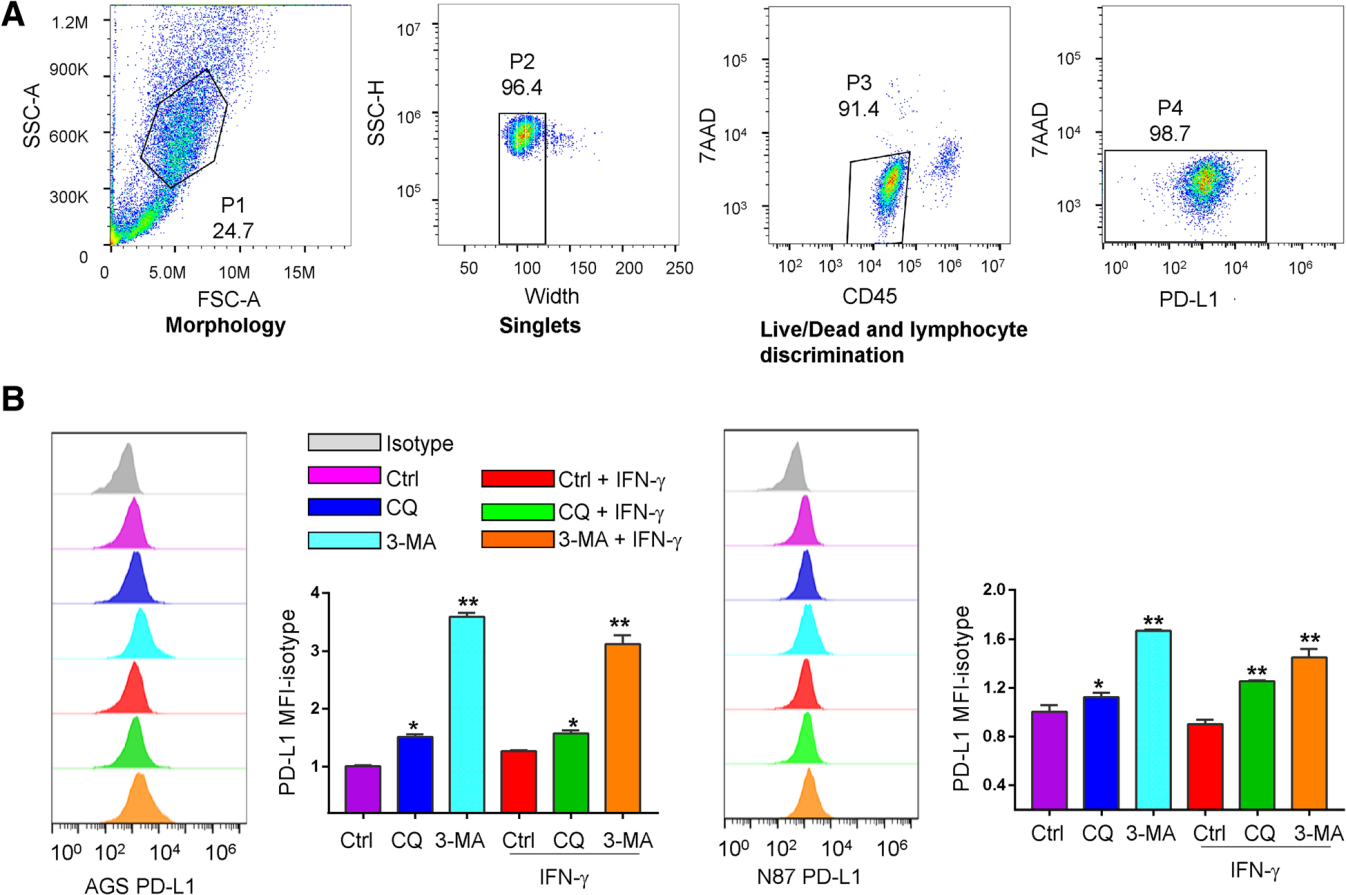
Inducing effct of lymphocytes and autophagy inhibitors on the expression of PD-L1 by gastric cancer cell lines. **a** Gating strategy of the cocultures. Gastric cancer cells were gated according to morphology (FSC-A vs. SSC-A) to single cell discrimination (SSC-W vs. SSC-A). The gastric cancer cells were then gated to perform live/dead and lymphocyte discrimination (CD45 vs. 7AAD). These cells were then checked for PD-L1 positivity (PD-L1 vs. 7AAD). The assays on each single sample were repeated at least 3 times. **b** Evaluation of the expression of PD-L1 in the gastric cancer cell lines AGS or NCI-n87 cocultured with lymphocytes in the presence of chloroquine or 3-MA. Control cells was off drugs for 3 days before the harvest for flowcytometry (Material and Methods). The ratio of PD-L1 MFI minus Isotype control was shown as mean ± S.D. relative to Ctrl from 3 independent experiments, *p < 0.05, **p < 0.01

In patients, lymphocytes very often infiltrate tumors and therefore tumor cells are directly exposed to the secreted cytokines. Our results so far indicated that expression of PD-L1 was increased in gastric cancer cells by autophagy inhibition (Figs. 2 and 3). Meanwhile, it has been reported that the autophagy inhibitor chloroquine inhibited human CD4^+^ T-cell activation and suppressed cytokine secretion, including tumor necrosis factor (TNF)-α, IFN-γ and interleukin (IL)-10 following anti-CD3/anti-CD28 activation [23], which may potentially inhibit the levels of PD-L1 expression in the adjacent tumor cells. To investigate the net effects of pharmacological inhibitors of autophagy on PD-L1 expression in gastric cancer cells in the presence of lymphocytes, an in vitro coculture experiment was set up where the cocultures of gastric cancer cells and lymphocytes were treated with either one of these drugs (Fig. 5a). Similar to our data presented in Fig. 2a, chloroquine or 3-MA treatment resulted in a significant induction of PD-L1 expression in the cocultured cells (Fig. 5b) (ANOVA *p* < 0.05).

### Autophagy markers were positively associated with PD-L1 in patients with gastric cancer

**Fig. 6:**
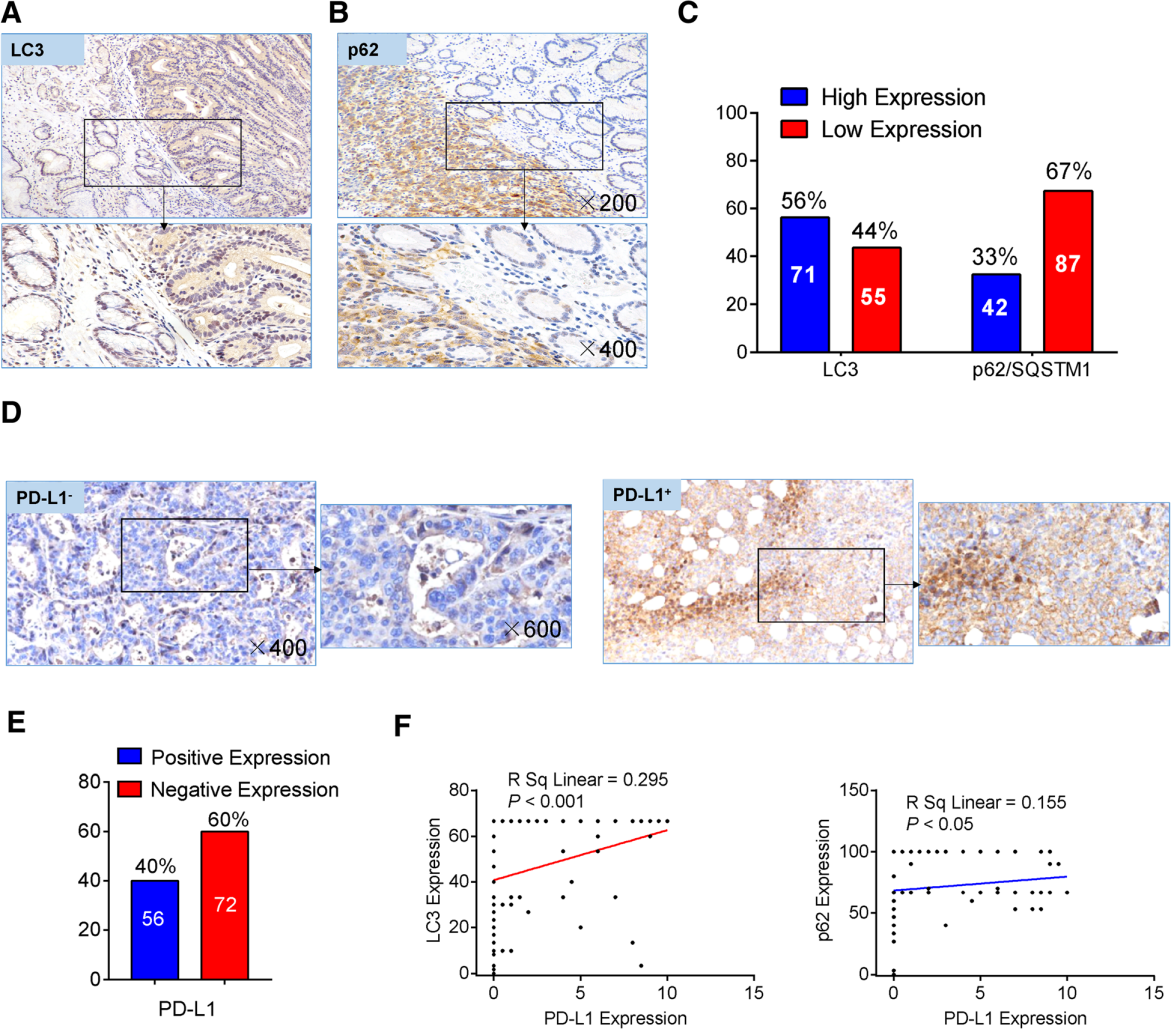
Immunohistochemical staining of PD-L1 and autophagy markers in human gastric cancers. **a** LC3 was found to be mainly expressed in the cytoplasm of gastric cancer cells. **b** p62/SQSTM1 staining was found in both the nucleus and cytoplasm. **c** p62/SQSTM1 and LC3 expression levels were dichotomized into high and low categories based upon total IHC expression score (4–9, high; 0–3, low). Missing data for p62/SQSTM1 (*n* = 8) and LC3 (*n* = 11). **d** PD-L1 protein was detected in patients with gastric cancer by IHC. Patients with ≥5% positive tumor cells or immune cells was considered to be PD-L1 positive. **e** The levels of PD-L1 expression were dichotomized into positive (≥ 5%) and negative categories (< 5%). **f** p62/SQSTM1 and LC3 expression levels were positively correlated with the levels of PD-L1 protein based upon total IHC expression score. Magnification, × 200, × 400, × 600

We further evaluated the clinicopathological and prognostic significance of autophagy markers, namely LC3B and p62/SQSTM1, in relation to PD-L1 in patients with gastric cancer. Marked expression of total LC3B and p62/SQSTM1 were found in cancer cells but not in surrounding stromal cells as revealed by immunohistochemistry. LC3 was mainly expressed in the cytoplasm of the cancer cells, while p62/SQSTM1 was expressed in both the nucleus and cytoplasm (Fig. 6a-b). Among 137 patients, LC3 and p62/SQSTM1 expression were positive in 42 (53%) and 71 (32%) patients, respectively (Fig. 6c). PD-L1 protein was mainly found in the cytoplasm and membrane of cancer cells (Fig. 6d). Of the 137 GC patients, 56 cases showed positive PD-L1 staining, among which 43 cases were patients with massive lymphocyte infiltration, and 81 cases showed negative PD-L1 staining (Fig. 6e). There was no correlation between expression of autophagy markers and clinicopathological features, such as age, gender, tumor site and pathological stage. However, as shown in Table 1, LC3 expression was significantly associated with lymphocyte infiltration. p62/SQSTM1 expression was found to be associated with Lauren histological type and lymphocyte infiltration in patients with gastric cancer. In addition, double staining analysis showed the co-expression of LC3, p62/SQSTM1 and PD-L1 (Additional file 1: Figure S6A-B). Importantly, we also found that there were statistically significant correlations among LC3, p62/SQSTM1 and PD-L1 expression levels (Fig. 6f, Additional file 1: Figure S6C and Table 2).

**Table 1:** Relationship between Autophagy markers and clinicopathological features in 137 gastric cancer cases

	LC3			p62/SQSTM1		
	High	Low		High	Low	
	(*n* = 71)	(*n* = 66)	*p* value	(*n* = 42)	(*n* = 95)	*p* value
Parameter						
Age						
≤ 59	34 (50%)	40 (50%)	0.161	28 (41.1%)	46 (58.9%)	0.045
> 59	37 (62.7%)	26 (37.2%)		14 (22.9%)	49 (77.1%)	
Gender						
Male	55 (57.2%)	46 (42.7%)	0.883	36 (37.9%)	65 (62.1%)	0.099
Female	16 (51.6%)	20 (48.3%)		6 (17.6%)	30 (82.3%)	
Tumor site						
Proximal	30 (62.5%)	20 (37.5%)	0.562	16 (33.3%)	34 (66.6%)	0.677
Antral	41 (51.9%)	46 (48.1%)		26 (32%)	61 (68%)	
TNM						
I-II	41 (48%)	39 (52%)	0.725	24 (24%)	56 (75%)	0.413
III-IV	30 (52.7%)	27 (47.3%)		18 (34%)	39 (66%)	
Lauren’s classification						
Intestinal-type	34 (60.7%)	26 (39.3%)	0.059	15 (26.7%)	45 (73.3)	0.013
Diffuse/mixed-type	37 (52.1%)	40 (47.9%)		27 (40%)	50 (60%)	
MLI						
Yes	33 (46.6%)	11 (53.3%)	0.000052	23 (19.4%)	70 (80.5%)	0.015
No	38 (69%)	55 (31%)		19 (51.9)	25 (48.1%)	

*MLI* Massive Lymphocyte Infiltrating

**Table 2:** Association of PD-L1 and autophagy-related proteins

Parameter	PD-L1		
	Positive	Negative	*p*-Value
	(*n* = 56)	(*n* = 81)	
LC3			
Negative	20 (30.3%)	46 (69.9%)	0.006
Positive	36 (50.7%)	35 (49.2%)	
p62/SQSTM1			
Negative	29 (30.5%)	66 (69.4%)	0.00012
Positive	27 (64.2%)	15 (35.7%)	

## Discussion

Autophagy has opposing, context-dependent roles in cancer and perturbations in autophagy are found in gastric cancer [24]. As one of the most important survival mechanisms, autophagy helps tumor cells to adjust and adapt to an unfavourable environment, to escape from immune surveillance, and hence to promote tumor growth. Recent studies have delineated the mechanism underlying autophagy and the intricate involvement of PD-L1/PD1 axis in cancer cells. A study by Clark et al. found that tumor-intrinsic PD-L1 signals regulate cell proliferation and autophagy in ovarian cancer and melanoma. Tumor cells with high levels of PD-L1 expression are more sensitive to autophagy inhibitors than cells with lower PD-L1 levels in murine melanoma cells and human ovarian cancer cells [14]. Melanoma cell-intrinsic PD-1 cooperates with PD-L1 to promote tumorigenesis and modulates downstream effectors of mTOR signaling [17]. Blockade of PD-L1 in sarcoma cells inhibits mTOR activity and dampens glycolysis, thereby restoring glucose in tumor microenvironment [25]. Depletion of glucose also induces autophagy through the mTOR complex 1 pathway [26]. Until now, the link between autophagy and the immune checkpoint molecule PD-L1 is not quite well understood in gastric cancer. Here, we demonstrated that inhibition of autophagy by pharmacological or RNA interference approach could induce the expression of PD-L1, unveiling the unreported intrinsic regulation of PD-L1 by autophagy.

As a ligand of PD-1, PD-L1 is a transmembrane protein that is expressed on a wide variety of cells including tumor cells to inhibit CD8^+^ T cell activities and suppress antitumor immunity. So, the PD-L1 protein on cell membrane mainly exerts its antitumor effect. Thus, we detected the expression of surface PD-L1 by flow cytometry in accordance with many of the published papers studying PD-L1 to determine its functional proportion [14, 15, 27–29]. Also, we evaluated the total expression of PD-L1 protein to demonstrate the upregulation of PD-L1 by autophagy inhibition through Western blots [30]. We demonstrated that blockade of autophagy increased the mRNA levels of PD-L1 as well as protein expression in gastric cancer cells. Accordantly, Yang et al. found that defective autophagy with deletion of *Atg5* by using a mouse model of cerulein-induced pancreatitis, activated the IκB kinase-related kinase TBK1 and promoted PD-L1 upregulation. These findings hinted at novel beneficial effects of autophagy inhibitors and their possible synergy with drugs targeting the PD-L1/PD-1 axis [16].

We noticed that the basal protein levels of PD-L1 are higher in NCI-N87 and AGS cells than other gastric cancer cells as shown in Fig. 1a, which may attribute to the specific genomic mutations harbored by the cells with *SMAD4* and *TP53* mutations in NCI-N87 cells and *CDH1, CTNNB1, KRAS* and *PIK3CA* mutations in AGS cells. In this regard, PD-L1 expression was significantly higher in tumors with *TP53* mutation in lung cancer while *KRAS* mutation could induce PD-L1 expression in lung adenocarcinoma [31, 32]. Also, the oncogenic activation of the AKT-mTOR pathway could upregulate the expression of PD-L1 in non-small cell lung cancer [33]. Both pharmacological agents and siRNA targeting non-lysosomal components of autophagy could up-regulate PD-L1 expression in gastric cancer cell lines, and the induction of IFN-γ further increased PD-L1 levels as shown in Figs. 2 and 3. 3-MA could effectively block an early stage of autophagy by inhibiting the class III PtdIns3K, but also non-selectively inhibit the class I PI3K and affect cell survival through AKT and other kinases which may in turn inhibit PD-L1 expression in particular settings. It is therefore likely that the overall upregulation of PD-L1 expression by autophagy inhibition is alleviated upon treatment by 3-MA compared to other autophagy inhibitors in AGS cells with *PIK3CA* mutations (Fig. 2b). Recently, the precise mechanism of how CQ blocks autophagy was firmly demonstrated – CQ mainly inhibits autophagy by impairing autophagosome fusion with lysosome but not affecting the acidity of this organelle [34]. The mutant p53 proteins was reported to counteract the formation of autophagic vesicles and the fusion with lysosomes via the repression of autophagy-related proteins and enzymes in pancreas and breast cancer cells [35]. Concordantly, we found that CQ induced a lower increase on PD-L1 expression compared to 3-MA and bafilomycin A1 group in NCI-N87 cells with *TP53* mutation (Fig. 2b). Thus, in studies where the effect of autophagy inhibition is being investigated, it is important to confirm results by inhibiting autophagy at different stages with several pharmacological inhibitors. We found that IFN-γ significantly induced PD-L1 expression through activation of STAT1 signaling independent of autophagy levels in AGS and NCI-n87 cells (Additional file 1: Figure S5A), which is in accordance with others in several types of cancer [36–38]. Upon autophagy inhibition, the levels of p-p65 was upregulated in AGS and NCI-N87 cells treated with or without IFN-γ (Additional file 1: Figure S5B). These results indicated that autophagy inhibition upregulated the levels of PD-L1 protein via NF-κB signaling whereas the IFN-γ induced PD-L1 expression through STAT1 signaling.

Clinical interventions to manipulate autophagy mainly by pharmacological inhibitors, including chloroquine and hydroxychloroquine, with other chemotherapeutics in search of synergistic interactions in cancer are already underway [39]. Due to the lack of our understanding of the interplay between autophagy and the immune response, a study has sought to elucidate their relationship and demonstrated that the antitumor adaptive immunity is not adversely impaired by autophagy inhibition in immune-competent mouse models of melanoma and mammary cancer [40]. Such findings are corroborated by our findings that autophagy inhibition had minimal effect on T cell function and PD-L1 levels were still upregulated in the cocultures of gastric cancer cells and PBMC (Fig. 5b). The increase in basal levels of PD-L1 expression in co-cultures makes the fold change reduced (Fig. 5b) compared to the gastric cancer cells alone group (Fig. 2a). The reduced fold change could be explained that the co-culture with lymphocytes itself already had an inducing effect on the expression of PD-L1 than cells without lymphocytes. In this respect, an inducing effect of lymphocytes on the expression of PD-L1 was found in cocultures with melanoma cells [29]. In addition, the pharmacological inhibitors may also have some effects on the lymphocytes, which has to be evaluated in our future study. In our study, we did not observe the inducing effect of IFN-γ on the levels of PD-L1 in co-cultured condition (Fig. 5b). The cytokines including IFN-γ secreted by the lymphocytes may have inducing effect on the levels of PD-L1 in co-culture conditions, which therefore attenuated the effect of exogeneous IFN-γ added to the co-cultures [41].

Our work suggests that autophagy inhibition plus anti-PD-L1 is an attractive combination for further investigation, particularly for tumors with high levels of autophagy, and provides potential biomarkers and mechanisms to assess clinical efficacy. However, contradictory evidence also exists in the literature. Peng et al. reported that the loss of PTEN decreased T cell infiltration in tumors, inhibited autophagy and was correlated with inferior outcomes with PD-1 inhibitor therapy [42]. PD-L1 expression can be induced by inflammatory cytokines or tumor-cell intrinsic signaling, including NF-κB, MAPK, PI3K, mTOR and JAK/STAT. Pearson correlation analysis as shown in Additional file 1: Figure S6C and Fig. 6f suggests that the expression of PD-L1 in gastric cancer is in part correlated with high levels of LC3 and p62/SQSTM1. These findings indicate that additional factors must be considered to discern the various scenarios in which blockade of autophagy would be beneficial in cancer therapy.

## Conclusions

Out data highlighted the existence of an additional, important signalling on the regulation of tumour intrinsic PD-L1. We found that inhibition of autophagy upregulated the expression of PD-L1 in gastric cancer cells in vitro and in vivo. As our understanding of tumor cell-intrinsic signals on the regulation of PD-L1 increases, our ability to predict treatment responses to various agents and combine them effectively will improve. We anticipate that our study will inform the development of autophagy inhibitors combined with immune checkpoint inhibitors in gastric cancer. Given the competing and context-dependent effects of autophagy, the best strategy would be to decide which patients would benefit from autophagy inhibition therapy.

## Additional file

Additional file 1: **Figure S1.** Flow cytometry histograms for PD-L1 expression of 8 gastric cancer cell lines. PD-L1 was expressed on 7.6% of AGS cells, 32.4% of NCI-N87 and 2.4% of SGC7901 cells. Less than 1% of BGC823, HGC27, MGC803, MKN45, SNU1 cells were detected to express PD-L1. **Figure S2.** LC3B positive puncta (green) was determined by immunofluorescence in AGS and NCI-n87 cells exposed to autophagy inhibitors, bafilomycin A1 (Baf, 10 nM) and chloroquine (CQ, 32 μM) for 24 h. Scale bar, 50 μm. **Figure S3.** The effect of chloroquine on the expression of PDL1 in in vivo subcutaneous xenograft models. **Figure S4.** (A) The effect of pharmacological inhibitors of autopahgy on the protein levels of p65, p-p65, IκBα, p-IκBα, IKKα/β and p-IKKα/β was detected by Western blots in AGS cells. **Figure S5.** (A) The protein levels of PD-L1, STAT1, p65 and p-p65 were detected by Western blots in AGS and NCI-n87 cells treated with IFN-γ for 24 h. **Figure S6.** (A) Representative images of double staining for LC3 and PD-L1. (DOC 5030 kb)

## Abbreviations

3-MA: 3-methyladenine
Baf: BafilomycinA1
BMS: BMS-345541
CQ: Chloroquine
Rap: Rapamycin
