# Supplementary material for: Autophagy inhibition enhances PD-L1 expression in gastric cancer
Source: J Exp Clin Cancer Res. 2019 Mar 29;38:140. doi: 10.1186/s13046-019-1148-5 (PMC6440013; doi:10.1186/s13046-019-1148-5)
Supplement: Supplementary file 1 — Figure S1. Flow cytometry histograms for PD-L1 expression of 8 gastric cancer cell lines. PD-L1 was expressed on 7.6% of AGS cells, 32.4% of NCI-N87 and 2.4% of SGC7901 cells. Less than 1% of BGC823, HGC27, MGC803, MKN45, SNU1 cells were detected to express PD-L1. Figure S2. LC3B positive puncta (green) was determined by immunofluorescence in AGS and NCI-n87 cells exposed to autophagy inhibitors, bafilomycin A1 (Baf, 10 nM) and chloroquine (CQ, 32 μM) for 24 h. Scale bar, 50 μm. Figure S3. The effect of chloroquine on the expression of PDL1 in in vivo subcutaneous xenograft models. Figure S4. (A) The effect of pharmacological inhibitors of autopahgy on the protein levels of p65, p-p65, IκBα, p-IκBα, IKKα/β and p-IKKα/β was detected by Western blots in AGS cells. Figure S5. (A) The protein levels of PD-L1, STAT1, p65 and p-p65 were detected by Western blots in AGS and NCI-n87 cells treated with IFN-γ for 24 h. Figure S6. (A) Representative images of double staining for LC3 and PD-L1. (DOC 5030 kb) [file 13046_2019_1148_MOESM1_ESM.doc]

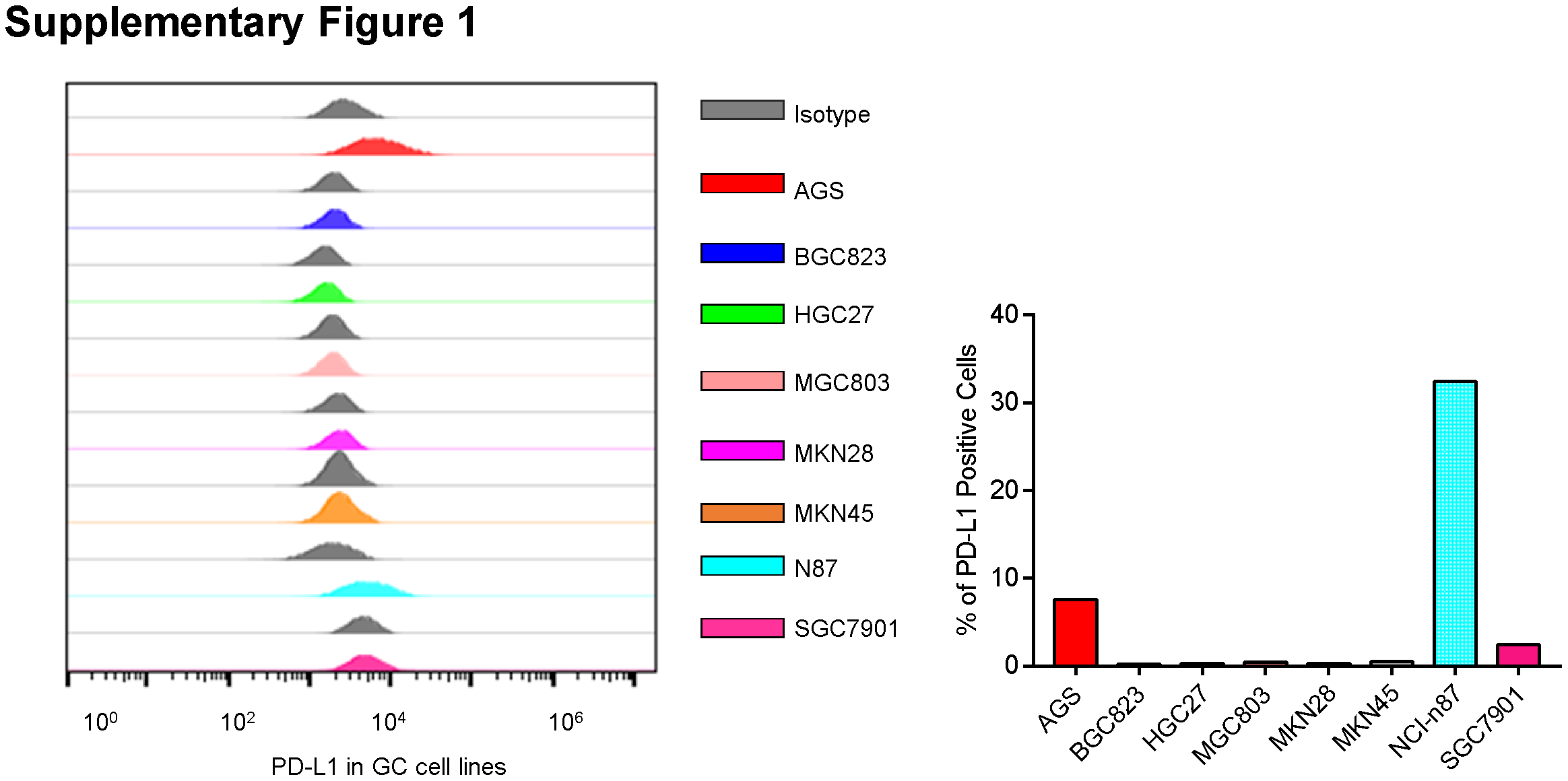
**Additional file 1**

**Figure S1.** Flow cytometry histograms for PD-L1 expression of 8 gastric cancer cell lines. PD-L1 was expressed on 7.6% of AGS cells, 32.4% of NCI-N87 and 2.4% of SGC7901 cells. Less than 1% of BGC823, HGC27, MGC803, MKN45, SNU1 cells were detected to express PD-L1.


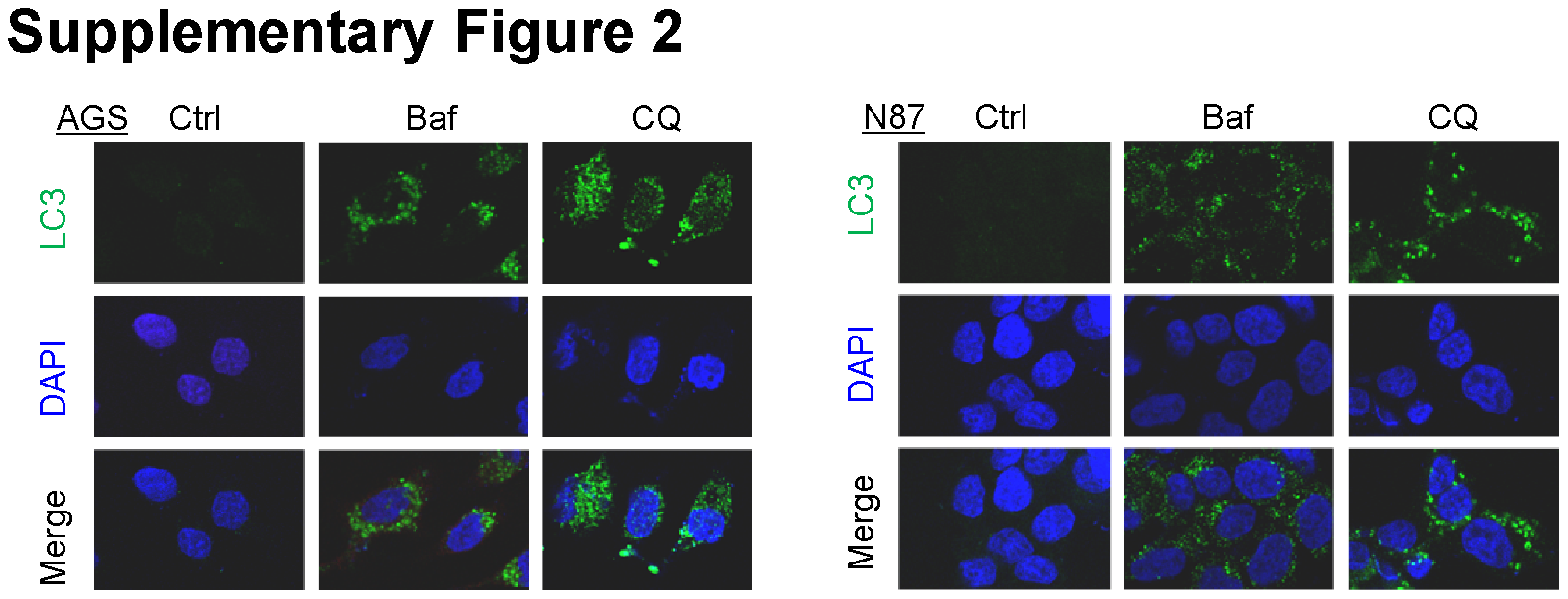


**Figure S2.** LC3B positive puncta (green) was determined by immunofluorescence in AGS and NCI-n87 cells exposed to autophagy inhibitors, bafilomycin A1 (Baf, 10 nM) and chloroquine (CQ, 32 μM) for 24 h. Scale bar, 50 μm.

**
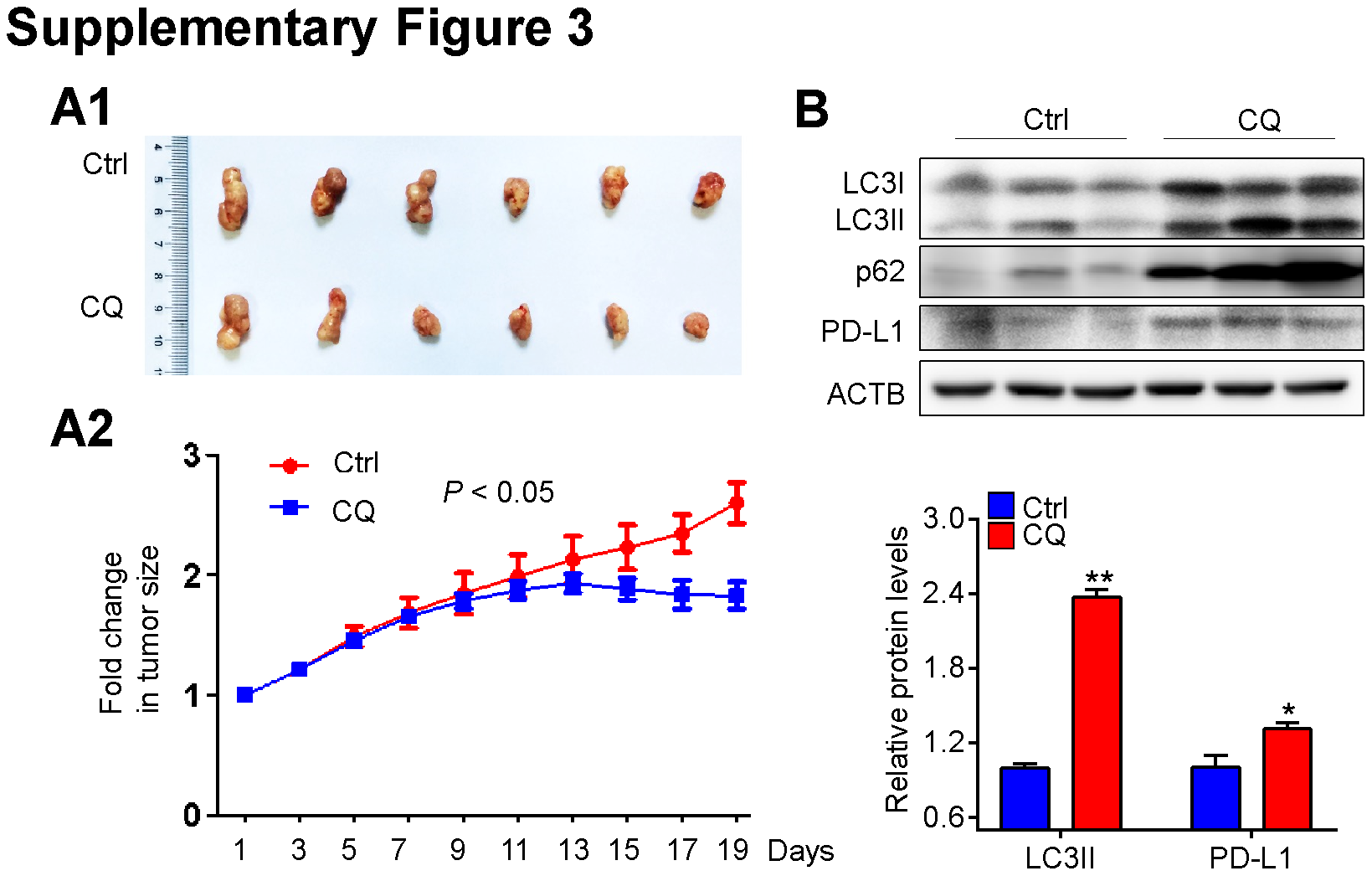
**

**Figure S3.** **The effect of chloroquine on the expression of PD-L1** **in *in vivo* subcutaneous xenograft models.** (**A1**) A representative image of tumour growth in nude mice subcutaneously inoculated with MKN45 cells received intraperitoneal injections of control or chloroquine (CQ, 50 mg/kg) every other day for 3 weeks. Tumour size was compared at the end of the experiment. (**A2**) Tumour growth cureve of CQ-injected mice was compared with vehicle treated mice. The data were mean ± SD (n = 6/group) of three separate experiments. (**B**) The expression of PD-L1 protein was significantly upregulated in CQ treated mice by Western blots. The inhibition of autophagy by CQ was evidenced by an increase in LC3-II/LC3-I ratio. Densitometry was performed with ImageJ. Quantification was shown as mean ± S.D (n = 6 per group); **p* < 0.05, ***p* < 0.01.

**
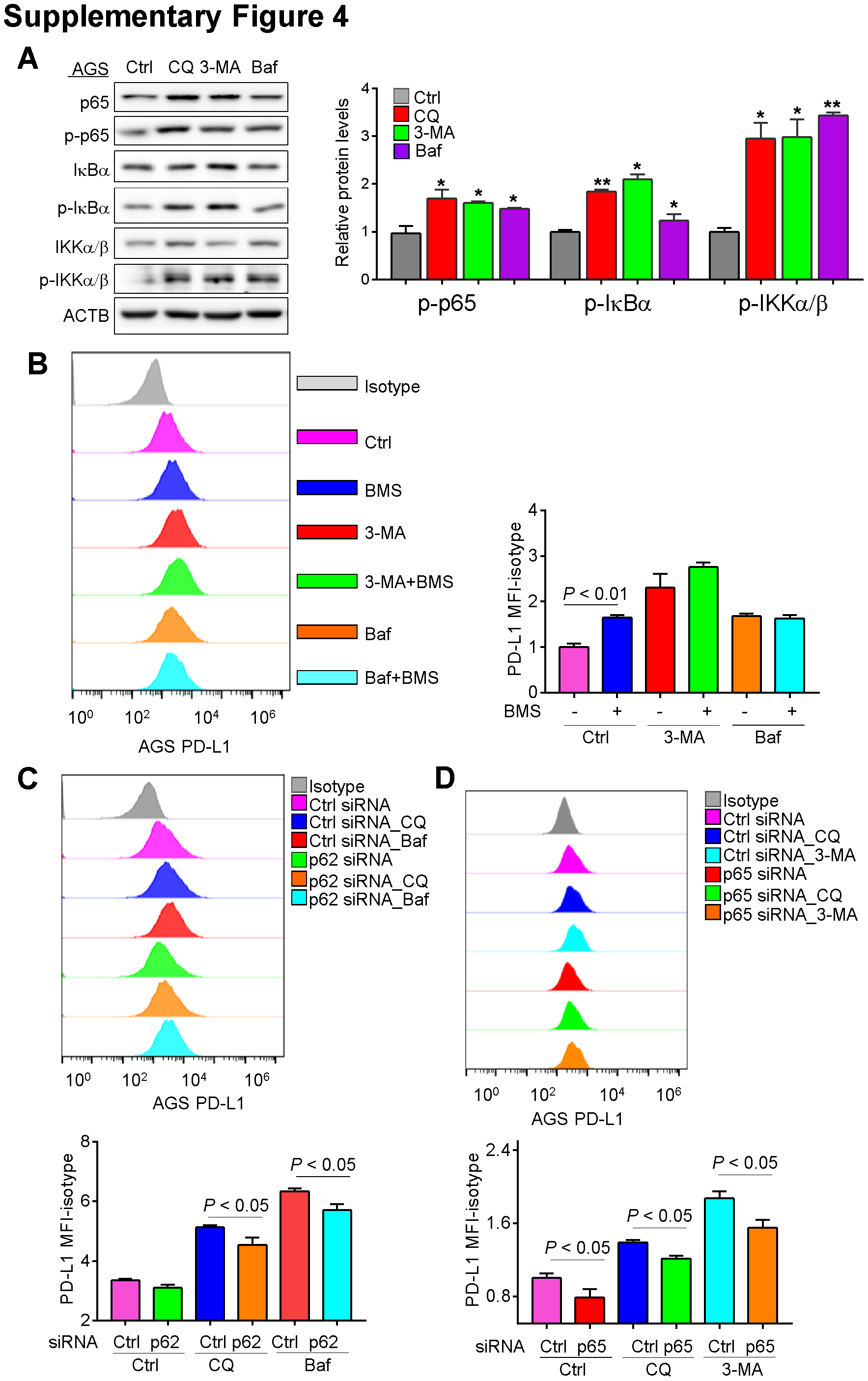
**

**Figure S4.** (**A**) The effect of pharmacological inhibitors of autopahgy on the protein levels of p65, p-p65, IκBα, p-IκBα, IKKα/β and p-IKKα/β was detected by Western blots in AGS cells. (**B**) The levels of PD-L1 protein were detected by flow cytometry in AGS cells upon autophagy inhibition after BMS-345541 cotreatment for 24h. (**C**) Knockdown of p62/SQSTM1 partially abolished the increase in PD-L1 protein induced by CQ and Baf in AGS cells as determined by flow cytometry. (**D**) Knockdown of p65 alleviated the induction of PD-L1 protein at 72 h post-transfection in AGS cells upon autophagy inhibition by flow cytometry. The ratio of PD-L1 MFI minus isotype control was shown as mean ± S.D. relative to Ctrl from 3 independent
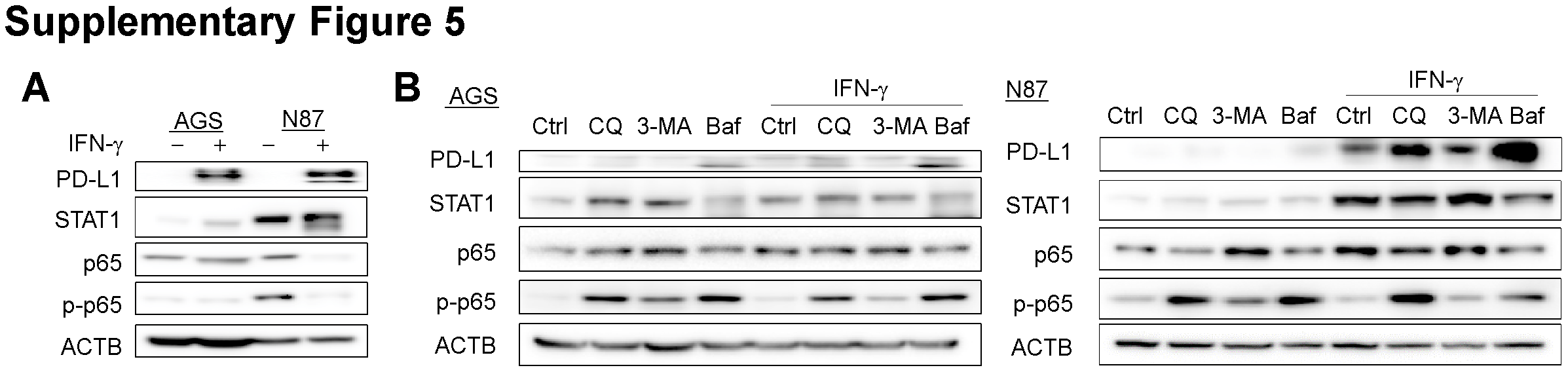
experiments.

**Figure S5.** (**A**) The protein levels of PD-L1, STAT1, p65 and p-p65 were detected by Western blots in AGS and NCI-n87 cells treated with IFN-γ for 24 h. (**B**) The levels of PD-L1, STAT1, p65 and p-p65 proteins were determined by Western blots in AGS and NCI-n87 cells treated with or without IFN-γ for 24 h under normal conditions or autophagy inhibition.


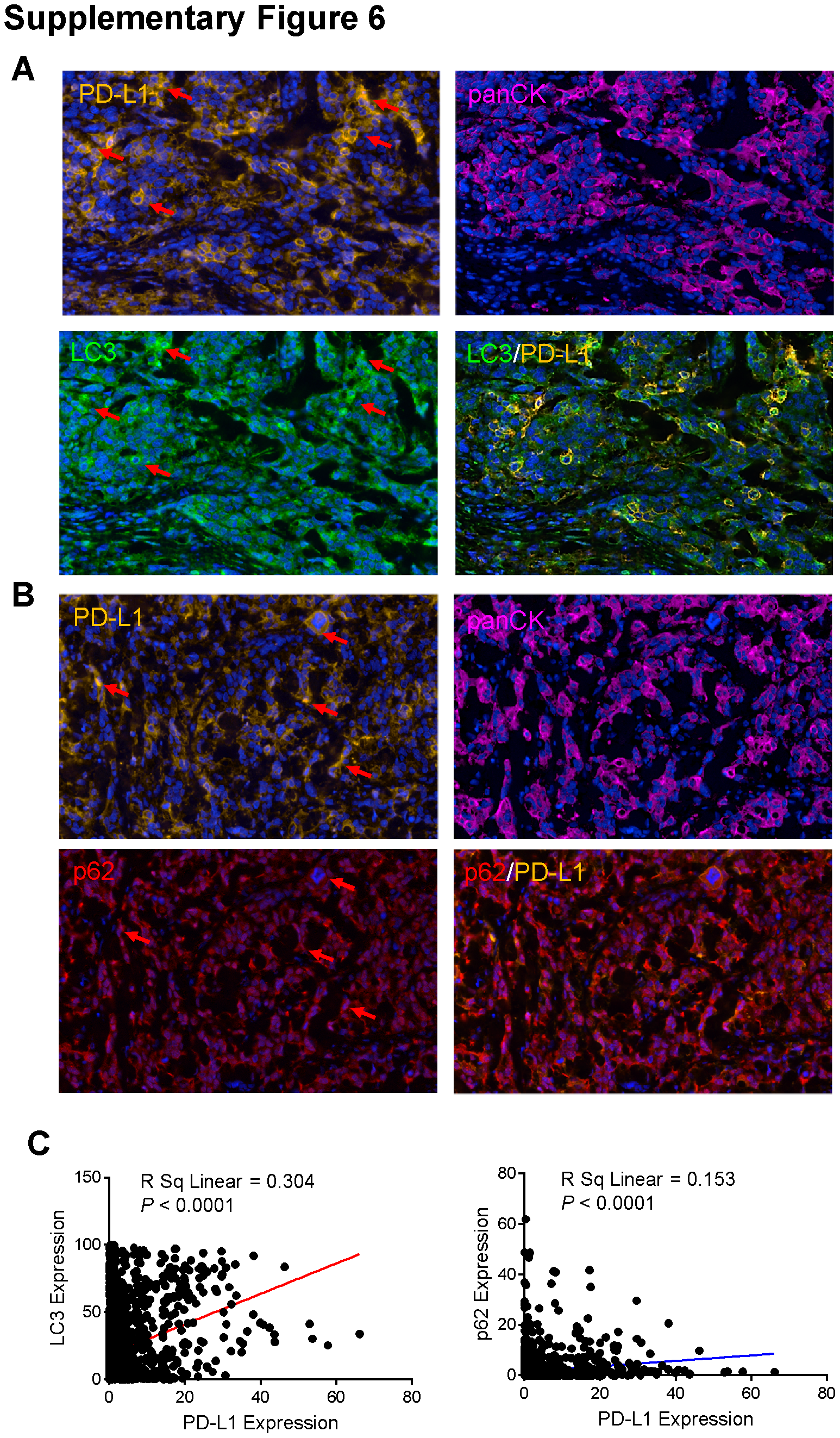


**Figure S6.** (**A**) Representative images of double staining for LC3 and PD-L1. (**B**) Representative images of double staining for p62/SQSTM1 and PD-L1. (**C**) Levels of LC3 and p62/SQSTM1 proteins correlated positively with levels of PD-L1 protein based upon total expression score in patients with gastric cancer. n= 105. Magnification, × 600.
